# Care Partner and Care-Receiver Kinship Influences Desire to Seek Long-Term Care After Hospitalization

**DOI:** 10.1093/geroni/igaf122.4082

**Published:** 2025-12-31

**Authors:** Ahmed-Rufai Yahaya, Azza A L Harrasi, Samiya AL-Hadhrami, Ashley Kuzmik, Liza Behrens, Diane Berish, Marie Boltz

**Affiliations:** The Pennsylvania State University, University Park, Pennsylvania, United States; The Pennsylvania State University, University Park, Pennsylvania, United States; The Pennsylvania State University, University Park, Pennsylvania, United States; Alzheimer’s Association, Chicago, Illinois, United States; The Pennsylvania State University, University Park, Pennsylvania, United States; The Pennsylvania State University, University Park, Pennsylvania, United States; The Pennsylvania State University, University Park, Pennsylvania, United States

## Abstract

Dementia affects 57 million people worldwide, and over 7 million Americans live with Alzheimer’s disease. Care partners play vital roles in providing care, but they also express several stressors, including behavioral symptoms, relationship stressors, and preparedness. There is evidence that the kinship (type of relationship) and quality of the care partner/care-receiver relationship are related to long-term care placement and quality of life. However, limited research has examined how kinship influences the desire to seek long-term care, post-acute hospitalization. This secondary analysis of a cluster randomized clinical trial (Family-centered Function-focused Care) examined the association between kinship and desire to seek long-term care for person with dementia at discharge, 2 months, and 6 months post-acute-hospitalization by care partners of hospitalized patients with dementia (N = 158). Care partner kinship type was classified as spouse/partner (n = 47), adult child (n = 86), or other relatives/friends (n = 25). Findings showed significant main effects of kinship type with a desire to seek long-term care [discharge covariates: F(2,158)=4.80, p=.010, partial-η²=.061; 2-month: F(2,145)=4.54, p=.012, partial-η²=.059; 6-month: F(2,145)=4.21, p=.017, partial-η²=.055] . Spouse/partner care partners showed lower desire to seek long-term care compared to adult children across timepoints (discharge: M = 0.96 vs. 1.34, p=.019; 2-month: M = 0.91 vs. 1.57, p=.013; 6-month: M = 0.66 vs. 1.55, p=.013) and others at 6-months (p=.002). Significantly older care partner age across all timepoints (p=.012, p=.004, p=.009), higher neuropsychiatric symptoms (p=.024), and lower care partner preparedness (p=.030) were associated with desire to seek long-term care. Our findings support interventions targeted at the care partner-recipient kinship type, aiming at preparedness and behavioral symptom management.

